# Differential recruitment efficacy of patient-derived amyloidogenic and myeloma light chain proteins by synthetic fibrils—A metric for predicting amyloid propensity

**DOI:** 10.1371/journal.pone.0174152

**Published:** 2017-03-28

**Authors:** Emily B. Martin, Angela Williams, Craig Wooliver, R. Eric Heidel, Sarah Adams, John Dunlap, Marina Ramirez-Alvarado, Luis M. Blancas-Mejia, Ronald H. Lands, Stephen J. Kennel, Jonathan S. Wall

**Affiliations:** 1 Department of Medicine, University of Tennessee Medical Center, Knoxville, Tennessee, United States of America; 2 Department of Surgery, University of Tennessee Medical Center, Knoxville, Tennessee, United States of America; 3 Microscopy Facility, University of Tennessee, Knoxville, Tennessee, United States of America; 4 Department of Biochemistry and Molecular Biology, and Immunology, Mayo Clinic, Rochester, Minnesota, United States of America; 5 Department of Radiology, University of Tennessee Medical Center, Knoxville, Tennessee, United States of America; University of Pittsburgh School of Medicine, UNITED STATES

## Abstract

**Background:**

Monoclonal free light chain (LC) proteins are present in the circulation of patients with immunoproliferative disorders such as light chain (AL) amyloidosis and multiple myeloma (MM). Light chain-associated amyloid is a complex pathology composed of proteinaceous fibrils and extracellular matrix proteins found in all patients with AL and in ~10–30% of patients who presented with MM. Amyloid deposits systemically in multiple organs and tissues leading to dysfunction and ultimately death. The overall survival of patients with amyloidosis is worse than for those with early stage MM.

**Methods and findings:**

We have developed a sensitive binding assay quantifying the recruitment of full length, patient-derived LC proteins by synthetic amyloid fibrils, as a method for studying their amyloidogenic potential. In a survey of eight urinary LC, both AL and MM-associated proteins were recruited by synthetic amyloid fibrils; however, AL-associated LC bound significantly more efficiently (p < 0.05) than did MM LCs. The LC proteins used in this study were isolated from urine and presumed to represent a surrogate of serum free light chains.

**Conclusion:**

The binding of LC to synthetic fibrils in this assay accurately differentiated LC with amyloidogenic propensity from MM LC that were not associated with clinical amyloid disease. Notably, the LC from a MM patient who subsequently developed amyloid behaved as an AL-associated protein in the assay, indicating the possibility for identifying MM patients at risk for developing amyloidosis based on the light chain recruitment efficacy. With this information, at risk patients can be monitored more closely for the development of amyloidosis, allowing timely administration of novel, amyloid-directed immunotherapies—this approach may improve the prognosis for these patients.

## Introduction

Monoclonal plasma cell proliferation is associated with a continuum of gammopathies characterized by the presence of a clonal plasma cell population in the bone marrow and the presence of intact monoclonal immunoglobulin and/or free light chain (LC) proteins in the serum [1–5]. In the US, the prevalence of monoclonal gammopathy of undetermined significance (MGUS), a pre-malignant state, is 4.2% in Caucasians over the age of 50, with 20% of those secreting only monoclonal light chain (LCMGUS) [6]. Longitudinal studies have demonstrated that LCMGUS precedes LC-associated multiple myeloma (MM) and that both conditions may lead to light chain-associated (AL) amyloidosis, a devastating protein misfolding disorder characterized by the systemic deposition of extracellular amyloid fibrils composed of LC proteins [7]. The genetic, biochemical and physiological factors that dictate which MGUS and MM patients will develop clinical LC amyloidosis are presently unknown. However, in addition to enigmatic host factors, the propensity of the monoclonal serum free light chain to aggregate into amyloid fibrils is a critically important factor [8, 9].

In contrast to patients with MM, 40% of AL patients have an abnormal serum free light chain ratio that may be detected as early as 11 years before diagnosis [10]. However, amyloidosis is usually diagnosed histologically much later in the course of the disease, relative to MM, by the presence of Congo red-birefringent deposits observed in bone marrow aspirates or subcutaneous fat pad biopsies The persistent accumulation of amyloid in peripheral organs, especially heart and kidneys, results in architectural damage and, possibly, the disruption of cellular metabolism and cytotoxicity [2, 11–13] which ultimately leads to progressive organ dysfunction and death. Overall, the median survival for AL patients is 3 y with a 5 y survival rate of approximately 30% [2], whereas American Cancer Society data indicates that the overall survival of stage I MM patients is 62 mos [14]. In patients with MM, only comorbidities of amyloidosis and renal impairment served as statistically significant independent prognostic factors that adversely affect patient survival [15]. Therefore, it is well established that the presence of monoclonal serum free light chains and their aggregation as amyloid fibrils is a significant clinical problem and contributes to morbidity and mortality in patients with plasma-cell related gammopathies such as MM.

Light chain amyloid deposits are most commonly composed of LC variable domain (VL) fragments [16, 17] yet the specific role of LC proteolysis and the exact nature of amyloid seed formation or recruitment *in vivo* remain largely undefined. Light chain fibril formation has been studied extensively *in vitro* through the use of recombinant VL fragments and principally indicates an inverse correlation between VL stability and the propensity for *in vitro* fibrillogenesis [18–21]. Despite our increased understanding of amyloid fibril formation and growth *in vitro*, the role of circulating intact LCs, in particular their potential recruitment by amyloid fibrils, has yet to be systematically elucidated. Moreover, the ability to accurately identify individuals with a monoclonal serum free LC component who are at risk of developing amyloidosis, due to unfavorable pro-amyloidogenic features of the LC, is severely limited. This is particularly troubling for patient populations that are at risk of developing amyloidosis, such as those with MM and the estimated 0.6 million Americans with LCMGUS.

To address these issues, we have developed a sensitive assay to study the recruitment of radiolabeled, patient-derived, urinary LC proteins *in vitro* using, as a template, pre-formed synthetic amyloid fibrils composed of a λ6 variable domain isolated from an AL patient Wil (rVλ6Wil; [21]). Our data indicate that, while rVλ6Wil fibrils recruited both MM and AL-derived LC proteins, the amount of protein recruited at 1 h, 3 h, and 24 h of incubation was significantly greater for the AL-associated proteins. The significant difference observed in the recruitment of AL and MM LC’s allowed discrimination of the patient groups. Such an assay may permit identification of those LCMGUS or MM patients at risk of developing LC amyloidosis during the course of their disease.

## Materials and methods

### Patient-derived and recombinant proteins

Monoclonal serum LC proteins were isolated from patients with an initial diagnosis of MM (n = 4) or AL amyloidosis (n = 3), or in one case, a patient initially presenting with MM who later developed LC amyloidosis (patient AL2κ; Table 1). These components were isolated from urine specimens as previously described [22]. The VL-subgroup classification was determined serologically using polyclonal antihuman VL-subgroup-specific antisera [22], or by amino acid sequence analysis [17]. Isolated LC proteins were lyophilized and stored at room temperature (RT) until used. Protein concentrations were measured by using a micro-bicinchoninic acid kit (Pierce, Rockford, IL). The rVλ6Wil protein was isolated from the periplasmic space of E. *coli* and purified by reverse phase high-pressure liquid chromatography, as previously described [23]. Integrity of the purified VL was determined by mass spectrometry using the calculated mass of 11,991. Fibrils of rVλ6Wil were generated by shaking protein (1 mg/mL) in sterile phosphate buffered saline, 150 mM NaCl, pH 7.2 at 37°C and 225 revolutions per min (C24 Incubator Shaker, New Brunswick Scientific, Edison, NJ) for ~72 h.

**Table 1:** Patient data and LC nomenclature.

AL patient-derived LC proteins				
Sample (historic nomenclature)	Sex/Age	LC isotype	Germline gene	References
AL1κ (CRO)	F/48	κ1	κI O1 (IGKV 1–39)	[24, 25]
AL2κ (HIG)	M/68	κ1	κ1 O18 (IGKV 1–33)	[25]
AL1λ (GIO)	M/37	λ6	λ6a (IGLV 6–57)	[26]
AL2λ (SUT)	M/72	λ6	λ6a (IGLV 6–57)	[26, 27]
**MM patient-derived LC proteins**				
MM1κ (GAL)	M/46	κ1	κ1 O18 (IGKV 1–33)	[28]
MM2κ (WAT)	M/81	κ1	κ1 O18 (IGKV 1–33)	[29]
MM1λ (HAG)	M/75	λ4b	λ4b (IGLV 4–69)	[30]
MM2λ (JRH)	M/73	λ3	λ3j (IGLV 3–9)	[31]

### SDS-polyacrylamide gel electrophoresis (SDS-PAGE) analysis

Five to 10 μg of each LC protein was added to 7.5 μL LDS sample loading buffer (4x; Invitrogen, Carlsbad, CA), and for reduced samples, 2.5 μL reducing buffer (10x; Invitrogen) was also added. All samples were boiled 10 min prior to loading onto 4–12% Bis-Tris SDS-PAGE gels (Invitrogen). Electrophoresis was performed using a Novex apparatus (ThermoFisher Scientific, Waltham, MA) with MES buffer at 200 V for 30 min. Gels were stained using 1% Coomassie (1% Brilliant blue R250, 50% methanol, 10% acetic acid) for 5 min followed by 15 min incubation in destaining solution (50% methanol/10% acetic acid) and then a water incubation for up to 24 hr.

### Antibodies

Antihuman total kappa light chain (14-6E4) and total lambda light chain (21-3F4) monoclonal antibodies [32] were used for the detection of intact light chain proteins via western blot analysis. In addition, antihuman free kappa light chain (LKC8) monoclonal antibody [32] was used for immunogold labeling.

### Western blot analysis

Proteins were transferred onto PVDF membranes (1 h, 30 V) from SDS-PAGE gels using the Novex system (ThermoFisher, Waltham, MA). After each outlined step, the blots were washed 3x using PBS with 0.05% tween-20 (PBST). The blots were blocked with 1% BSA (w/v) in PBS for 1 h on an orbital shaker prior to the addition of 10 mL of anti-total human κ monoclonal antibody (mAb, 14-6E4) or anti-total human λ (mAb 21-3F4) at 1 μg/mL in 1% BSA/PBST. Primary mAb was incubated for 1 h at RT on an orbital shaker before addition of 10 mL of a 1:5000 dilution of horse radish peroxidase-conjugated goat anti-mouse mAb (Jackson ImmunoResearch, West Grove, PA). After a 1 h incubation at RT, the blots were developed using ImmPACT diaminobenzidene peroxidase substrate (Vector Laboratories, Burlingame, CA).

### Surface plasmon resonance

Recombinant Vλ6Wil fibrils [21] were immobilized to a CM-5 chip using the amino-coupling method and reagents supplied with the BIAcore X instrument (GE Healthcare, Pittsburgh, PA). Briefly, chips were activated by injection (35 μL) of a mixture of N-ethyl-N’-(dimethylaminopropyl)carbodiimide (EDC) and N-hydroxysuccinimide (NHS) at a flow rate of 5 μL/min. A suspension of rVλ6Wil fibril, diluted to 100 μg/mL in pH 4.0 NaOAc buffer (35 μL), was probe sonicated (Tekmar Sonic Disrupter with microprobe) for 10 sec immediately before injection. After fibril coupling to the Fc-1 channel, the remaining active groups on the chip were blocked by injection of 35 μL of 1 M ethanolamine-HCl, pH 8.5. Non-fibrillar Vλ6Wil, which served as a control, was similarly coupled to a chip in the Fc-2 channel. An initial regeneration step consisting of a 20 μL injection of pH 1.5 glycine buffer with 1 M NaCl was performed and the baseline allowed to equilibrate for 30 min. Light chain preparations were diluted to a stock of 5 μg/mL in HBS-EP buffer. The LC was injected (50 μL) and the binding sensorgram collected for 450 sec. A 600 sec delayed wash cycle and an additional 600 sec lag was included in the sensorgram to facilitate binding profile analysis. The chip was subjected to a regeneration step and 30 min equilibration before the next test injection. Data were extracted from the sensorgrams and analyzed using BIAevaluation (v. 3) software.

### Light chain radiolabeling

Light chain proteins (50 μg) were added to 10 μL 0.5 M NaPO_4_ (pH 7.6) and radioiodinated with ~0.5 mCi of iodine-125 (^125^I; PerkinElmer, Waltham, MA) using 40 μg chloramine T, followed by addition of 40 μg sodium metabisulfite to quench the reaction. The radiolabeled product was diluted into 0.1% gelatin in sterile PBS and purified by gel filtration using a 5 mL-volume Sephadex G-25 column (PD10, GE Healthcare, Piscataway, NJ) equilibrated with 0.1% gelatin/PBS. Peak fractions of radioactivity were pooled, and the product’s radiochemical purity was measured by SDS-PAGE analyzed by phosphor imaging (Cyclone Storage Phosphor System; PerkinElmer).

### Solution phase, pulldown binding assays

Binding assays were conducted as previously described [33] with minor modifications. Briefly, Twenty-five μg of synthetic rVλ6Wil fibrils or control substrates (polystyrene beads, mouse liver homogenate, Aβ(1–40) fibrils or 25 μg monomeric LC protein) were suspended in 200 μL of PBST. Ten microliters (~10 ng) of radioiodinated protein was added and the suspension rotated at RT for 1 h, 3 h, or 24 h. The samples were then centrifuged at 15,000 × g for 10 min, supernatants collected and the pellets resuspended in 200 μL PBST before a second centrifugation at 15,000 × g for 10 min. Supernatants were again removed and pooled, and the pellets resuspended in 400 μL of PBST. The radioactivity in each supernatant and pellet was measured using a Cobra II gamma counter (PerkinElmer) with a 1 min acquisition. The percentage of ^125^I-LC in the pellet was determined as follows: Bound = [*Pellet* cpm/ (*Pellet* cpm + *Supernatant* cpm)] x 100. For competition assays, 100- or 1000-fold molar excess of competitor protein was added to the mixture containing fibrils and ^125^I-labeled rVλ6Wil prior to the 24 h incubation.

### Immunogold-labeling of AL1κ bound to rVλ6Wil fibrils

Twenty-five μg of rVλ6Wil fibrils were mixed with 1 μg AL1κ LC in 200 μL of tris-buffered saline (TBS) with 0.05% tween-20 (TBST) in a 1.5 mL-volume microcentrifuge tube. The sample was rotated at RT for 24 h before being centrifuged at 20,000 × g for 8 min and the supernatant was discarded. The pellet was washed in 400 μL TBST twice by centrifugation, before the sample was split into two 200 μL-volume samples in TBST. To one sample, 10 μg each of two biotinylated anti-κ mAbs (LKC8 and 14-6E4) were added; no antibodies were added to the other sample. Both samples were rotated 2 h at RT prior to two washes by centrifugation, as before. The pellets were resuspended in 200 μL TBST with addition of a 1:4 dilution of streptavidin/gold stock solution (10 nM-diameter; Electron Microscopy Sciences, Hatfield, PA). After an additional 2 h rotation at RT, four washes were performed to remove unbound streptavidin-gold. Both samples were resuspended in 50 μL TBST and stored at 4°C until being imaged.

### Electron microscopy

Five μL of fibril sample was applied to a freshly glow-discharged carbon film supported by a 200 mesh copper grid. After one min, the excess material was removed and the remaining sample was stained with one drop of 1% (w/v) uranyl acetate solution for one min. The grid was then washed with one drop of distilled water, dried, and examined in Zeiss Libra 200MC electron microscope (Peabody, MA). Images were recorded using an Ultrascan camera (Gatan Inc., Pleasanton, CA).

### Gel analysis of urea-treated fibrils

The pellet from an ^125^I-LC recruitment experiment was resuspended in 100 μL of 6 M urea in PBST. Samples were then rotated for 4 days at RT after which time 20 μL of sample was mixed with 7.5 μL SDS-PAGE loading buffer, boiled for 10 min and loaded onto 4–12% Bis-Tris SDS-PAGE gels (Invitrogen). The SDS-PAGE gel was then analyzed by a phosphor imager (Cyclone Storage Phosphor System; PerkinElmer) after 2 h exposure. Protein band profiles and peak intensity measurements (DLU; digital light units) were obtained using Optiquant software (v. 5.0; PerkinElmer). To ensure the urea treatment did not adversely affect the LC, control samples of LC alone in 6 M urea were also prepared and subjected to the same analysis as described above.

### Thermodynamic folding stability studies

Scan rate dependent thermal unfolding measurements were performed as previously described [34, 35]. Since the thermal unfolding of full length LC proteins is irreversible and kinetically controlled, the apparent transition midpoint (*Tm*_*app*_) depends on the applied scan rate and, therefore, is entirely determined by the kinetics of the formation of the final state. This circumstance makes a straightforward thermodynamic analysis, assuming equilibrium between states, impossible. Consequently, it was not possible to obtain meaningful thermodynamic parameters such as an equilibrium *Tm* or *ΔH* to describe the unfolding/refolding process. However, by using a fully kinetic two-state model, explicitly considering the rate constant for irreversibility, stability parameters were obtained using the data treatment described by [35].

### Statistical methods

Frequency and descriptive statistics were performed to check for coding errors and to meet statistical assumptions. Skewness and kurtosis statistics were used to test for normality. Any skewness or kurtosis statistic above an absolute value of 2.0 was assumed to be non-normal. Levene’s test of equality of variances was used to test the assumption of homogeneity of variance. In the event of a violation of a statistical assumption, non-parametric Mann-Whitney U tests were employed. Between-subjects comparisons were conducted using independent samples t-tests. Means and standard deviations are reported for parametric statistics and medians and interquartile ranges are reported for non-parametric tests. Bivariate correlations were used to assess the relationship between continuous and ordinal variables. Statistical significance was assumed at an alpha value of 0.05. All analyses were conducted using SPSS (v. 22; IBM Corporations, Armonk, NY).

### Study approval

This study was approved by the Institutional Review Board (IRB) at the University of Tennessee Medical Center. The IRB determined that the research qualified for exemption based upon 45 CFR 46.101 (b).

## Results

### SDS-PAGE and western analyses of light chain proteins

**Fig 1:**
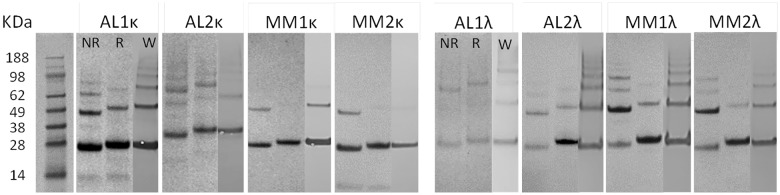
Analyses of AL or MM patient-derived LCs by SDS-PAGE and western blot. Electrophoresis was performed under non-reducing (NR) and reducing (R) conditions. Western blotting (W) was performed using reduced protein samples with a panel of anti-κ and or λ LC mAbs. Monomeric and dimeric LC appeared at ~ 24 KDa and 48 KDa, respectively.

Light chain protein preparations, isolated from patient’s urine, were evaluated using both SDS-PAGE, under reduced (R) and non-reduced (NR) conditions, and western blot (W) analyses (Fig 1). Monomeric LC proteins were present with a molecular weight of ~24 kDa, with the exception of AL2κ which has been previously shown to be glycosylated in the VL domain. All stained protein bands in the gel were detected in western blots using anti-total κ (14-6E4) or anti-total λ (21-3F4) mAbs that bind epitopes located in the LC constant domain. The presence of varying amounts of LC dimer was observed at ~48 kD. SDS-stable multimers (>48 kD) were observed principally in the ALκ and MMλ patient samples. In all cases, the protein bands in the Coomassie-stained gel corresponded to LC-associated bands in the western blots (Fig 1). Despite using 10 μg of LC protein in each lane, protein AL1λ appeared faintly in the gel; however, all protein bands were positive using the anti-total λ LC mAb. A low molecular weight species (~14 kDa) in proteins AL1κ and MM2κ was observed that did not appear on the western blot, possibly indicating the presence of free VL domain fragments.

### Recruitment of proteins by rVλ6Wil fibrils

**Fig 2:**
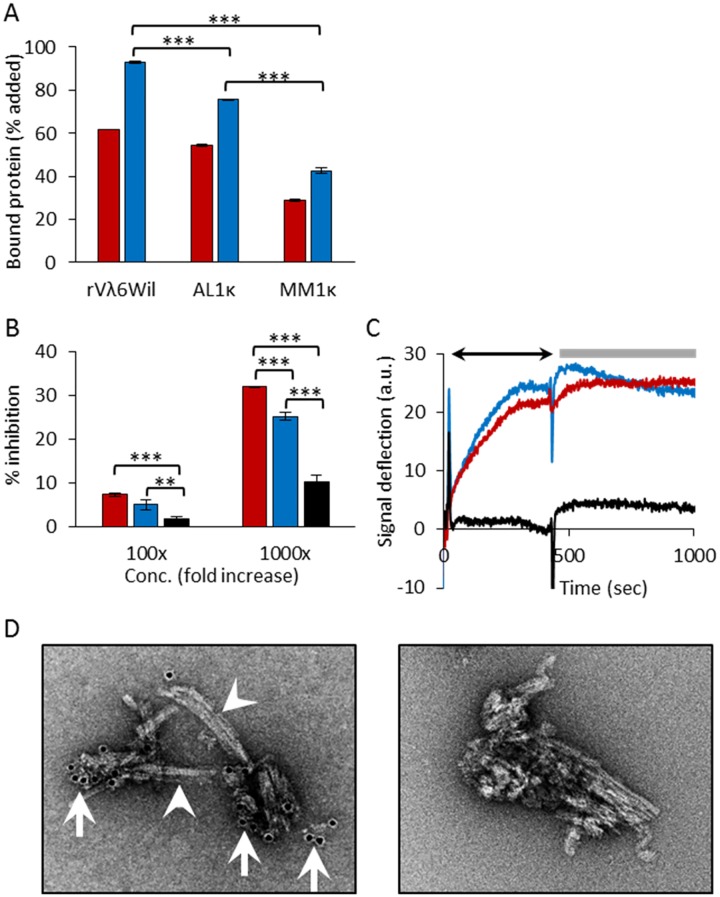
Recruitment of VL domains and LC proteins by rVλ6Wil fibrils. (A) The recruitment (% bound) of ^125^I-labeled rVλ6Wil, AL1κ and MM1κ assessed using a pulldown assay after 1 h (red) or 24 h (blue) incubation. Mean ± SD (*n* = 2 or 3). (B) Inhibition of ^125^I-rVλ6Wil binding to rVλ6Wil fibrils by the presence of a 100- or 1000-fold molar excess non-radiolabeled rVλ6Wil (red), AL1κ (blue) or MM1κ (black). Mean ± SD (*n* = 3). (C) Recruitment of LC and VL by rVλ6Wil fibrils measured by surface plasmon resonance. Representative sensorgrams for the binding of rVλ6Wil (red), AL1κ (blue) and MM1κ (black) showing binding (arrow) and washout phases (bar). (D) Immunogold electron micrographs of AL1κ recruited by rVλ6Wil fibrils. Left panel, 10 nm-diameter gold particles (arrows) indicate the presence of the anti-κ mAb (binding AL1κ) on the fibrils (arrowheads). Right panel, shows control sample with no anti-κ LC mAb added. In A and B, data were analyzed by ANOVA with a Bonferroni post hoc test for multiple comparisons (**, *p* < 0.001; ***, *p* < 0.0001).

To establish and validate the recruitment pulldown assay, we first analyzed the binding of ^125^I-labeled soluble rVλ6Wil protein by homologous rVλ6Wil synthetic amyloid fibrils (Fig 2A). The recruitment of ^125^I-rVλ6Wil monomer into fibrils increased from 62% to 93% at 1 h to 24 h of incubation (Fig 2A). Using this same assay, we next investigated whether the fibrils would support heterologous recruitment of radiolabeled LC using the AL- and MM-associated proteins AL1κ and MM1κ, respectively. Both proteins were recruited; however, at 24 h, the binding of AL1κ (76%) was significantly greater (*p* < 0.0001) than that of MM1κ (43%; Fig 2A).

To demonstrate that the interaction of LC AL1κ and MM1κ with the rVλ6Wil fibrils was similar to homologous rVλ6Wil binding, we utilized a competition assay wherein the recruitment of ^125^I-rVλ6Wil by fibrils was measured in the presence of a 100- or 1000-molar excess of non-radiolabeled rVλ6Wil (red), AL1κ (blue) or MM1κ (black) LC (Fig 2B). A 1000-fold molar excess of unlabeled rVλ6Wil inhibited binding by 30%. In contrast, inhibition of ^125^I-rVλ6Wil by AL1κ and MM1κ was less efficient at 25% and 10%, respectively (Fig 2B). Inhibition of ^125^I-rVλ6Wil recruitment by AL1κ was significantly greater than that seen using MM1κ protein (*p* < 0.0001). To address whether radiolabeled rVλ6Wil, AL1κ or MM1κ proteins bound non-specifically to materials and surfaces other than synthetic amyloid fibrils or if they became aggregated under the assay conditions, we performed binding assays using a variety of control substrates including polystyrene amino-derivatized particles, healthy mouse liver homogenate, monomeric precursor protein, or the pulldown procedure with no particulate target substrate present. In all cases, the rVλ6Wil and MM1κ LC showed no binding (<0.2%); however, the AL1κ LC gave values of ~5.0% (Table 2). While the non-specific pulldown of AL1κ was relatively high, it was still a minor component of the recruitment values when rVλ6Wil fibrils were used as substrate. In addition, we examined the binding of ^125^I-labeled AL1κ and MM1κ to 25 μg of Aβ(1–40) synthetic fibrils, which may have served as a negative fibril control. However, due to the cross-seeding phenomenon, following a 24 h incubation, AL1κ and MM1κ bound to these fibrils at 69% and 52%, respectively, indicating that non-AL related synthetic fibrils cannot serve as negative controls in this assay system.

**Table 2:** Binding of ^125^I-labeled proteins to non-fibrillar substrates in a pulldown assay.

Test Protein	Substrate	Recruitment (mean % ± SD)
^125^I-rVλ6Wil	25 μg WIL monomer	0.02 ± 0.014
	~ 25 μg polystyrene amino particles	0.01 ± 0.014
	25 μg WT mouse liver homogenate	0.4 ± 0.050
	no substrate	0.02 ± 0.021
^125^I-AL1κ	25 μg AL1κ monomer	4.9 ± 0.311
	~ 25 μg polystyrene amino particles	4.04 ± 0.163
	25 μg WT mouse liver homogenate	5.47 ± 0.014
	no substrate	4.03 ± 0.516
^125^I-MM1κ	25 μg MM1κ monomer	0.07 ± 0.042
	~ 25 μg polystyrene amino particles	0.01 ± 0.014
	25 μg WT mouse liver homogenate	0.81 ± 0.042
	no substrate	0.32 ± 0.00

We next investigated the recruitment of rVλ6Wil, AL1κ and MM1κ LC by rVλ6Wil fibrils using an alternative binding assay, surface plasmon resonance, in which protein was administered at a constant flow rate over a chip coated with rVλ6Wil fibrils. A parallel channel coated with monomeric rVλ6Wil served as the negative (background) control. The binding profiles for rVλ6Wil (red) and AL1κ (blue) were similar, with a slow binding phase over 450 sec (arrow), resulting in a signal deflection of ~ 23 a.u. (Fig 2C). During the 600 sec washout phase (bar), neither rVλ6Wil nor AL1κ protein dissociated from the fibril substrate. In contrast to these two AL-related proteins, MM1κ LC (black) showed no binding to the fibrils under these conditions (Fig 2C).

### Electron microscopic analysis of rVλ6Wil fibrils with recruited AL1κ LC

Immunogold electron microscopy was used to visualize the binding of AL1κ LC to rVλ6Wil fibrils (Fig 2D). The distribution of AL1κ LC (evidenced by the presence of anti-κ LC mAb-bound 10 nm-diameter gold particles; arrows) was generally at the ends of discrete aggregates of rVλ6Wil fibrils and less commonly observed along the length of the fibril (arrowheads; Fig 2D left panel). A sample of fibrils with AL1κ LC bound, but with no mAb added, served as a non-specific binding control. No gold particles were observed associated with the majority of fibrils in control preparations (Fig 2D right panel).

### Recruitment of AL and MM LC proteins by rVλ6Wil fibrils

**Fig 3:**
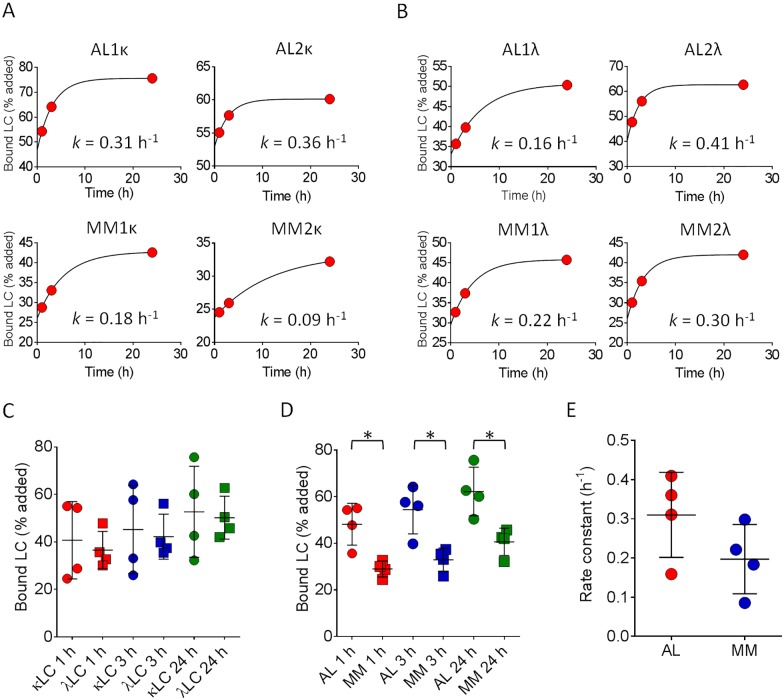
Kinetic recruitment of LC by rVλ6Wil fibrils. Recruitment of ^125^I-labeled AL and MM κ (A) or λ (B) LC proteins by rVλ6Wil fibrils (each point represents the mean of n = 2 observations, error bars are not visible). Rates were calculated using a single exponential binding equation, R^2^ for each fit was > 0.95. (C) Binding of ^125^I-labeled κ (circle) and λ (square) LC by rVλ6Wil fibrils after 1 h (red), 3 h (blue) or 24 h (green) of incubation. (D) Recruitment of AL- (circle) and MM-associated (square) LC was significantly different at 1 h (red), 3 h (blue) or 24 h (green) of incubation (*, *p* < 0.05). (E) Calculated recruitment rates of AL (red) and MM (blue) LC were not significantly different.

In our initial experiments, both the AL1κ and MM1κ LC were recruited by rVλ6Wil fibrils but with significantly different efficacy. Therefore, to discern whether this was a general disease-related phenomenon or unique to the individual monoclonal proteins, we expanded our study to include six more radiolabeled LC proteins (Table 1, Fig 3A & 3B). All radioiodinated LC proteins were recruited by rVλ6Wil fibrils, regardless of the isotype or the disease association, and binding of each LC increased over the 24 h of incubation. When comparing the LC isotype as a factor governing recruitment competency, we observed no significant difference between the binding of κ and λ LC to fibrils at 1 h, 3 h, or 24 h of incubation (Fig 3C). However, when the LC recruitment data were grouped in terms of disease state, AL vs MM, significantly more AL-associated LC bound (*p* < 0.05) to the rVλ6Wil fibrils at every time point measured (Fig 3D). Notably, the AL2κ LC, which was derived from a patient who presented with MM and later developed hepatosplenic LC amyloidosis, behaved like other AL-derived LC with 60% bound at 24 h (mean for all 4 AL LC = 62%) and with the second fastest estimated rate of recruitment, 0.36 h^-1^. In general, the calculated rate of AL LC binding was variable but > 0.3 h^-1^ (mean = 0.31 ± 0.1), which contrasted with the MM proteins that were more slowly sequestered by the fibrils (mean = 0.20 ± 0.1); however, the mean rate of recruitment of ^125^I-labeled AL LCs was not significantly faster than that for MM proteins likely due to the small sample size (Fig 3E; *p* = 0.09).

**Fig 4:**
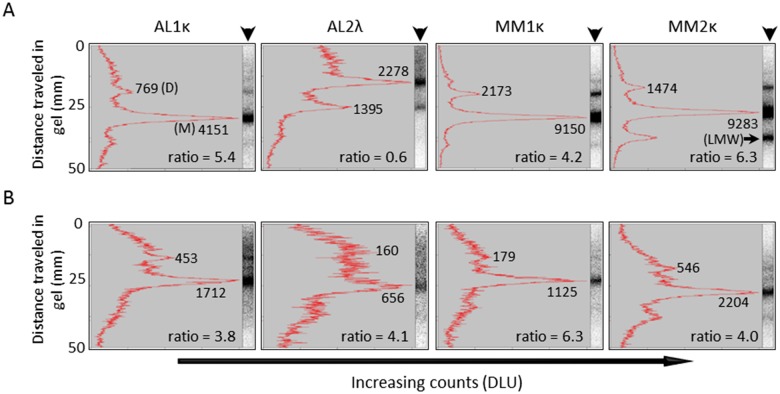
Monomeric LC proteins are preferentially sequestered by rVλ6Wil fibrils. (A) Quantitative densitometry of representative reduced SDS-PAGE gel electrophoretic profiles (arrowhead) of urea-treated ^125^I-labeled LC. (B) Analysis of urea-treated rVλ6Wil fibrils following recruitment of ^125^I-labeled LC. The monomer:dimer ratio was calculated for each LC. Where: DLU, digital light units; M, monomer; D, dimer; LMW, low molecular weight component.

We next sought to discern the structural species (i.e., monomer, dimer, multimer, or low molecular weight fragment) in the radiolabeled polydisperse LC preparation that were preferentially recruited by rVλ6Wil fibrils. The ^125^I-labeled LC was isolated from the fibrils following a recruitment assay by incubating the complex in 6 M urea for 72 h. A sample of urea-treated, non-recruited ^125^I-LC stock served as a control. Treatment of the ^125^I-labeled LC protein did not significantly alter the LC profile as determined by gel electrophoresis (S1 Fig). Following SDS-PAGE under reducing conditions, the gel profiles (arrowheads) were quantitatively analyzed by phosphor screen imaging and densitometry (Fig 4). The radiolabeled starting material was predominantly monomer (M) and dimer (D), with varying amounts of multimers and low molecular weight (LMW) fragments (Fig 4A). The monomer:dimer ratio was generally >4, with the exception of AL2λ, which contained ~2-fold more SDS-stable non-covalent dimers as compared to momomeric LC. The ^125^I-LC components isolated from the fibril complex following a 24 h recruitment assay were similarly evaluated (Fig 4B). In most cases, the monomeric LC was preferentially associated with the rVλ6Wil fibrils (see e.g., AL2κ, Fig 4B); however, for certain LCs, notably AL1κ, the dimer was also observed associated with the fibril preparation, as evidenced by the change in monomer:dimer ratio from 5.4 to 3.8 in the starting material and fibril preparation, respectively. In no case was there evidence of preferential recruitment of a low molecular weight fragment if one was present in the LC stock solution (see e.g., MM2κ, Fig 4B). These data are consistent with the preferential heterologous recruitment of whole LC by the rVλ6Wil fibrils. Furthermore, the concentration of the monomeric LC fraction in the radiolabeled preparation (based on analysis of the gel electrophoresis) does not correlate strictly with the binding efficacy, as measured in the pulldown assay.

**Fig 5:**
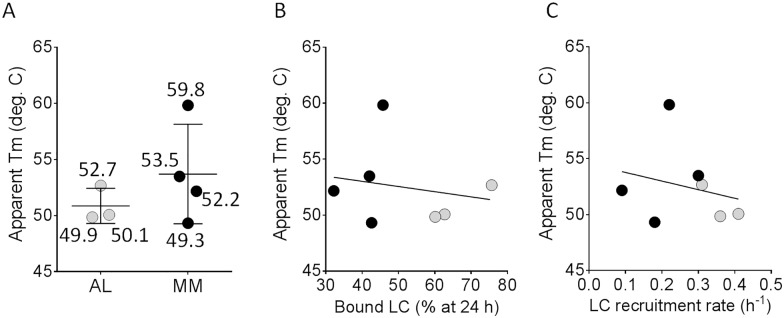
Thermodynamic folding stability of AL- and MM-associated LC does not correlate with fibril recruitment efficacy. (A) Estimates of the thermodynamic folding stability expressed as the apparent melting midpoint at a scan rate of 0.5°C/min (*Tm*_*app*_). Numbers indicate the *Tm*_*app*_ of individual LCs. Correlation analyses of (B), *Tm*_*app*_ and rVλ6Wil fibril recruitment efficacy at 24 h of incubation (% bound) and of (C), *Tm*_*app*_ and the calculated recruitment rate. Black and gray symbols in B and C represent the MM- and AL-associated LC, respectively. Correlation coefficients in B and C were not significant (*p* = 0.68).

The thermodynamic folding stability, expressed as apparent Tm (*Tm*_*app*_), of the LC proteins was estimated by performing scan rate-dependent thermal unfolding measurements using CD spectroscopy to monitor changes in secondary structure as a function of temperature. The AL1λ LC was insoluble at the concentration required for this analysis; therefore, this protein was omitted from the analyses (Fig 5). When the remaining seven LC proteins were grouped and analyzed together, the mean *Tm*_*app*_ for AL proteins (51°C) was only three degrees lower than that for the MM protein group (54°C; Fig 5A). Additionally, the monomeric component observed in preparations of AL2κ, MM1κ and MM1λ was purified and the *Tm*_*app*_ of this material was shown to be within error of the *Tmapp* values obtained from the polydisperse material (data not shown). This difference was not significant. Furthermore, there was no significant correlation between the folding stability (*Tm*_*app*_) and either the recruitment efficacy at 24 h (Fig 5B) or the calculated rate of recruitment (Fig 5C).

## Discussion

Our theoretical understanding of LC amyloidogenesis comes almost exclusively from *in vitro* studies of fibrillogenesis using recombinant VL fragments of κ4 [20, 36–39], λ6 [21, 40, 41] and κ1[18, 19, 42–44] LC subgroups produced in E. *coli*. Fibril formation from these components often requires harsh conditions such as low pH, agitation, or the presence of chaotropes, indicating the need for a denaturing milieu to initiate protein misfolding and, consequently, fibril formation. Although there are rare exceptions [19, 45], these studies demonstrated a general inverse correlation between VL folding free energy and the propensity for *in vitro* fibrillogenesis, such that less stably folded VL domains more readily form fibrils. Thus, *de novo* fibrillogenesis from soluble VL domains can be rapid and requires misfolding of the VL, allowing structured self-association into thermodynamically stable multimers that act as templates, or seeds, for the recruitment of additional VL domains. Template-mediated seeding is a universal paradigm in amyloid-related pathologies that underlies the proposed transmissibility of amyloid diseases [46–50].

In patients with AL amyloidosis, fibrils are most commonly composed of the VL with a small number of constant domain amino acids as evidenced from amino acid sequencing of peptides isolated from amyloid extracts [16, 17]. Additionally, mass spectrometric [51], immunohistochemical [52] and immunoblotting techniques [53] have shown the presence of LC constant domain fragments, indicating the likely presence of full length LC; however, the amount cannot be quantified and the presence of polyclonal as well as monoclonal proteins cannot be ruled out. Of particular import is the role of circulating free LC proteins in amyloid fibril growth. It remains unclear whether the LC proteins undergo proteolysis prior to incorporation into the fibril leading to VL-VL interactions or if, alternatively, amyloid fibrils grow via the interaction of intact LC proteins, some of which undergo proteolytic cleavage of the LC constant domain, following recruitment into the fibril [52]. Furthermore, it is unknown whether, *in vivo*, the initial formation of an amyloid seed and the subsequent process of fibril elongation, or growth, involves the same LC species. i.e., VL or full length protein.

Protein instability and misfolding are critical factors associated with the formation of the initial fibril seed from VL domains; however, the importance of these factors in governing the role of full length LC in amyloidosis *in vivo* remains enigmatic. The presence of the LC constant domain in the full length LC modulates the *de novo* fibrillogenesis of LC proteins *in vitro*, and under physiological conditions, full length light chains do not form thioflavin T positive fibrils as readily as recombinant VL domains [53–55]. Our data show that neither the rate of recruitment nor the amount of protein recruited correlated with the stability of the LC protein, expressed as *Tm*_*app*_. Furthermore, our data indicate that full length LC proteins can be effectively recruited by an excess of pre-formed, heterologous fibrils. Consistent with this, studies of recombinant λ6 VL domains have found no correlation between the thermodynamic stability of the protein and the rate of fibril growth when an excess of seed is present [40]. These findings indicate a possible mechanistic difference between the initial formation of the stable multimeric seed and the subsequent fibril elongation phases of amyloidosis.

In the presence of preformed fibrils composed of VL fragments, similar to those found in the amyloid deposits of patients, heterogeneous LC proteins can be recruited almost as effectively as the homologous VL (Figs 2A & 3D). However, no LC proteins were recruited as efficiently as the homologous VL, possibly due to sub-optimal binding of the heterologous proteins and the presence of the constant domain, which may cause steric obstruction–a phenomenon previously observed [47]. Our data indicate that both AL and MM LC interact at sites on the amyloid fibril similar to those occupied by homologous rVλ6Wil precursor during fibril elongation, as evidenced by their ability to compete for the recruitment of rVλ6Wil precursor. However, AL-related LC proteins inhibited rVλ6Wil recruitment significantly more effectively than did MM patient-derived LC. This may indicate that AL LC proteins have a greater affinity for the rVλ6Wil fibrils as compared to the MM proteins, but this is not likely related to the folding stability of the LC as it did not correlate with the LC *Tm*_*app*_ (Fig 5). Efficient recruitment of heterologous LC proteins by the rVλ6Wil fibrils suggests that LC amyloid deposits in patients might be capable of binding polyclonal free LC which are ubiquitously present at 3–26 μg/mL in the circulation [56].

Although synthetic rVλ6Wil fibrils recruited both AL and MM patient-derived LC, the amount of MM LC recruited per 24 h was ~50% less than that for AL-associated LC. The differential recruitment of MM and AL LC by rVλ6Wil fibrils mimics the competition data discussed above and was not explained by differences in the protein folding stability, supporting the paradigm that protein misfolding is not required for effective LC recruitment and fibril elongation and further suggesting a kinetic component of this recruitment. By analogy to Aβ peptide fibril elongation, LC proteins may undergo a “dock and lock” mechanism [57] in which initial LC recruitment (docking) occurs with the protein in a native state which may be followed by stable (irreversible) incorporation of the LC into the fibril. One implication of such a template-induced misfolding model for LC is that the process may expose hitherto cryptic epitopes and protease sites during the “locking” process. This may allow facile cleavage of constant domain fragments, by e.g. cathepsin-like enzymes [58] or permit immunological recognition by amyloid fibril-specific antibodies [59–64].

Using the fibril recruitment assay, we were able to demonstrate the binding of patient-derived, full length LC proteins by synthetic amyloid fibrils composed of variable domain fragments. Moreover, based on the recruitment competency, this assay was able to distinguish AL and MM-associated LC with a high degree of significance (*p* < 0.005), based on two different criteria: (i) the extent of binding to rVλ6Wil fibril seeds at 1 h, 3 h and 24 h of incubation and (ii) the ability of MM and AL LC proteins to inhibit recruitment of ^125^I-rVλ6Wil by the fibrils.

It is noteworthy that the difference between recruitment efficacy of AL and MM-derived proteins is not great in the pulldown assay, which indicates that MM-derived LC proteins are not “benign” and are capable of binding to amyloid fibrils under appropriate conditions. The data shown in Fig 3D suggest the possibility that all LC proteins have the potential to be recruited by a fibrillar template. In concert with this, we acknowledge that monoclonal LC proteins in patients with MM cannot be definitively determined to be unassociated with amyloid in the patient since occult amyloid deposits in patients with MM may not be evident, and autopsies are not routinely performed to demonstrate the absence of amyloid in MM patients. Consequently, our data imply that there is a continuum of recruitment propensity (or amyloid risk) for all LC proteins. Given this interpretation of the data, we speculate that an assay measuring directly, or indirectly, the recruitment efficacy of LC might be developed to identify subjects with MGUS, smoldering myeloma, or MM with a serum free LC component who may be at increased risk for developing amyloidosis. In this study, the AL2κ LC was obtained from a patient originally diagnosed in 1992 with MM but who, ~36 months later, developed symptomatic hepatic, splenic and renal LC amyloidosis. In the fibril-binding assay, this LC protein was recruited more efficiently by synthetic rVλ6Wil fibril, as compared to the other MM patient-derived LCs, and behaved similar to AL-associated LC proteins (60% bound at 24 h).

With respect to the development of a clinically relevant and practical assay to measure recruitment competency of serum free light chains, there is much to consider, and the present pulldown assay format is unlikely to be suitable due to the need for LC-isolation, radiolabeling etc. In this regard, we have considered further developing an SPR-based technique capable of differentiating patient-derived LC proteins. Although such an assay appears feasible based on the data presented in Fig 2C, at present, SPR assays are not routinely performed on serum samples due to their complex composition. Therefore, an effective SPR-based recruitment assay likely requires purification of the LC from the serum sample which is cumbersome, and the SPR assay itself requires a high degree of technical expertise. Given these considerations, we are developing an assay that indirectly measures LC recruitment efficacy that may be amenable to direct evaluation of serum samples in a 96-well microplate and does not require radioactive manipulations.

In addition to the recruitment efficacy of the LC protein, other criteria such as the serum free LC concentration [65] or LC germline gene usage [1], might be incorporated into an “amyloid risk” index that may provide a sensitive method for predicting the potential for amyloid disease. Given the documented relationship between decreased folding stability of the light chain variable domain and enhanced fibrillogenicity, inclusion of such a measurement in an amyloid propensity index would be desirable; however, given the complexity of performing such an analysis for every patient, this approach is impractical in the clinical setting. Identifying patients likely to develop LC amyloid may prompt closer clinical surveillance of those at risk, leading to earlier detection of amyloid and timely therapeutic intervention with novel therapies that are currently being developed [59, 66–68]. Early recognition and appropriate intervention are critical to maintaining organ function and, thus, prolonged patient survival. Further work to validate the clinical utility of a LC protein recruitment assay is underway.

## Supporting information

S1 Fig Electrophoretic analysis of urea-treated LC protiens. Urea-treatment of AL and MM-derived LC proteins does not significantly alter the LC monomer-dimer relationship as evidenced by SDS gel electrophoresis followed by autoradiographic analysis.
(DOCX)
