# Supplementary material for: Differential recruitment efficacy of patient-derived amyloidogenic and myeloma light chain proteins by synthetic fibrils—A metric for predicting amyloid propensity
Source: PLoS One. 2017 Mar 28;12(3):e0174152. doi: 10.1371/journal.pone.0174152 (PMC5369765; doi:10.1371/journal.pone.0174152)
Supplement: S1 Fig — Urea-treatment of AL and MM-derived LC proteins does not significantly alter the LC monomer-dimer relationship as evidenced by SDS gel electrophoresis followed by autoradiographic analysis. (DOCX) [file pone.0174152.s001.docx]

PONE-D-16-34736 Supplemental information

Figure S1. Urea-treatment of AL and MM-derived LC proteins does not significantly alter the LC monomer-dimer relationship as evidenced by SDS gel electrophoresis followed by autoradiographic analysis.
